# Diagonal Earlobe Crease as a Significant Marker for Coronary Artery Disease: A Case-control Study

**DOI:** 10.7759/cureus.1013

**Published:** 2017-02-05

**Authors:** Rida Kamal, Komal Kausar, Ahmed H Qavi, Moeed H Minto, Fariha Ilyas, Salman Assad, Saeed U Shah

**Affiliations:** 1 Department of Medicine, Shifa College of Medicine, Islamabad, Pakistan; 2 Department of Medicine, Montefiore New Rochelle Hospital, Albert Einstein College of Medicine, New Rochelle, NY, USA; 3 Department of Medicine, Eastbourne District General Hospital, UK; 4 Department of Medicine, University of Texas at Austin, Dell Medical School, Austin, TX, USA; 5 Department of Medicine, Shifa Tameer-e-Millat University, Islamabad, Pakistan; 6 Department of Cardiology, Shifa College of Medicine, Islamabad, Pakistan

**Keywords:** diagonal earlobe crease, coronary artery disease, cad, delc, hypertension, diabetes, smoking

## Abstract

**Objectives:**

To investigate the association between diagonal earlobe crease (DELC) and coronary artery disease (CAD). Limited data exists in South Asia and no prior studies have been performed in Pakistan to assess this relationship.

**Methods:**

In this case-control study, 200 participants from December 2015 to March 2016 at Shifa International Hospital, Islamabad, Pakistan were enrolled. Consecutive non-probability sampling was used to recruit patients. Cases were enrolled from cardiac care unit (CCU) of the hospital with angiography-proven CAD. Controls were selected from surgical, medical and neurology units of the hospital if they had no previously established evidence or symptoms of CAD. Patients were evaluated in terms of age and any history of hypertension, diabetes and/or smoking. Cases and controls were examined separately by two investigators for the unilateral or bilateral presence of DELC of the lobular portion of either auricle. Patients with ear piercings were excluded from the study. The data was analyzed in statistical product and service solutions (SPSS) (IBM, Delaware, Chicago), and an online statistical software.

**Results:**

Out of the 200 patients, 126 (63%) were males and 74 (37%) were females. In the 100 cases, 76 had DELC and 24 had no crease whereas, among the 100 controls, 36 had DELC and 64 had no DELC (p <0.001, OR = 5.63, CI = 2.91-10.93). The prevalence of diseases such as hypertension, diabetes, smoking among the cases and controls were 66%, 53%, 27% and 27%, 18%, 25% respectively. The effect of hypertension and diabetes on the presence of DELC was statistically significant (p <0.05) but the impact of smoking on DELC presence was insignificant (p >0.05).

**Conclusion:**

There is a significant association between DELC and CAD. This is the first case-control study from South Asia disclosing this important correlation. Our study also reports a high frequency of DELC in patients suffering from hypertension and diabetes mellitus. No association between smoking and DELC was found.

## Introduction

Cardiovascular diseases, including heart disease and stroke, are the world’s largest killers, claiming 17.5 million lives a year globally [1]. In Pakistan, coronary heart disease claims about 200,000 lives per year [2]. The situation is alarming as the number is consistently on the rise. Efforts for early diagnosis of coronary artery disease (CAD) have compelled many clinicians to look for noninvasive markers of disease. Over the years, diagonal earlobe crease (DELC) has been regarded as a controversial physical sign in predicting the occurrence of CAD. DELC runs from the lower pole of the external meatus, diagonally backward to the edge of the lobe at approximately 45 degrees. In 1973, Frank first reported the association of the presence of DELC with CAD. It was deemed as the Frank sign [3]. However, a consensus for the routine use of DELC in CAD patients is yet to be formed. Furthermore, there is no data available to assess this association in the South Asian population, which suffers from an increasingly significant burden of CAD, diabetes mellitus and hypertension. There is also a need to carry out further studies in various populations to observe this influence of ethnicity and conclude that this association is applicable to all races [4-9].

The earlobe is supplied by end arteries without a possibility of collateral circulation. Thus, the postulated theory suggests that any pathological condition influencing the microvasculature such as CAD, diabetes, and hypertension may contribute to the formation of diagonal earlobe crease. Moreover, diffuse loss of elastin and elastic fibers were observed in biopsy specimens taken from earlobe creases depicting the vasculature morphology present in the coronary bed, pathognomonic of CAD [4]. This is, to our knowledge the first case-control study conducted in South Asia to investigate the association of CAD and DELC. This study was done on Pakistan population aiming to assess the potential use of DELC as a marker to predict the risk of development of CAD. Informed consent was obtained from the patient for this study.

## Materials and methods

This study was performed after approval by the institution’s review board and patients’ informed consents. It is a case-control study, conducted on male and female aged between 25-75 years at a tertiary-care hospital, Shifa International Hospital (SIH), Islamabad, Pakistan. The sample size was calculated through an online software, Raosoft with 95% confidence interval. A total of 200 participants were investigated, 100 cases and 100 controls. Consecutive non-probability sampling technique was used to recruit patients in this study. Cases were enrolled from cardiac care unit of SIH with proven CAD through angiographic results from December 2015 to March 2016. Controls were selected if they had no previously established evidence of CAD or symptoms of cardiovascular disease such as dyspnea upon exertion and chest pain. They were enrolled from surgical, medical and neurology units of the hospital. We evaluated patients in terms of gender, history of hypertension, diabetes mellitus and smoking habits. Patients were considered hypertensive according to Joint National Committee-seven (JNC) criteria. Diabetes was documented if the patient was on insulin or an oral hypoglycemic. Smokers were defined by a history of cigarette smoking during that time period or prior to the study.

**Figure 1:**
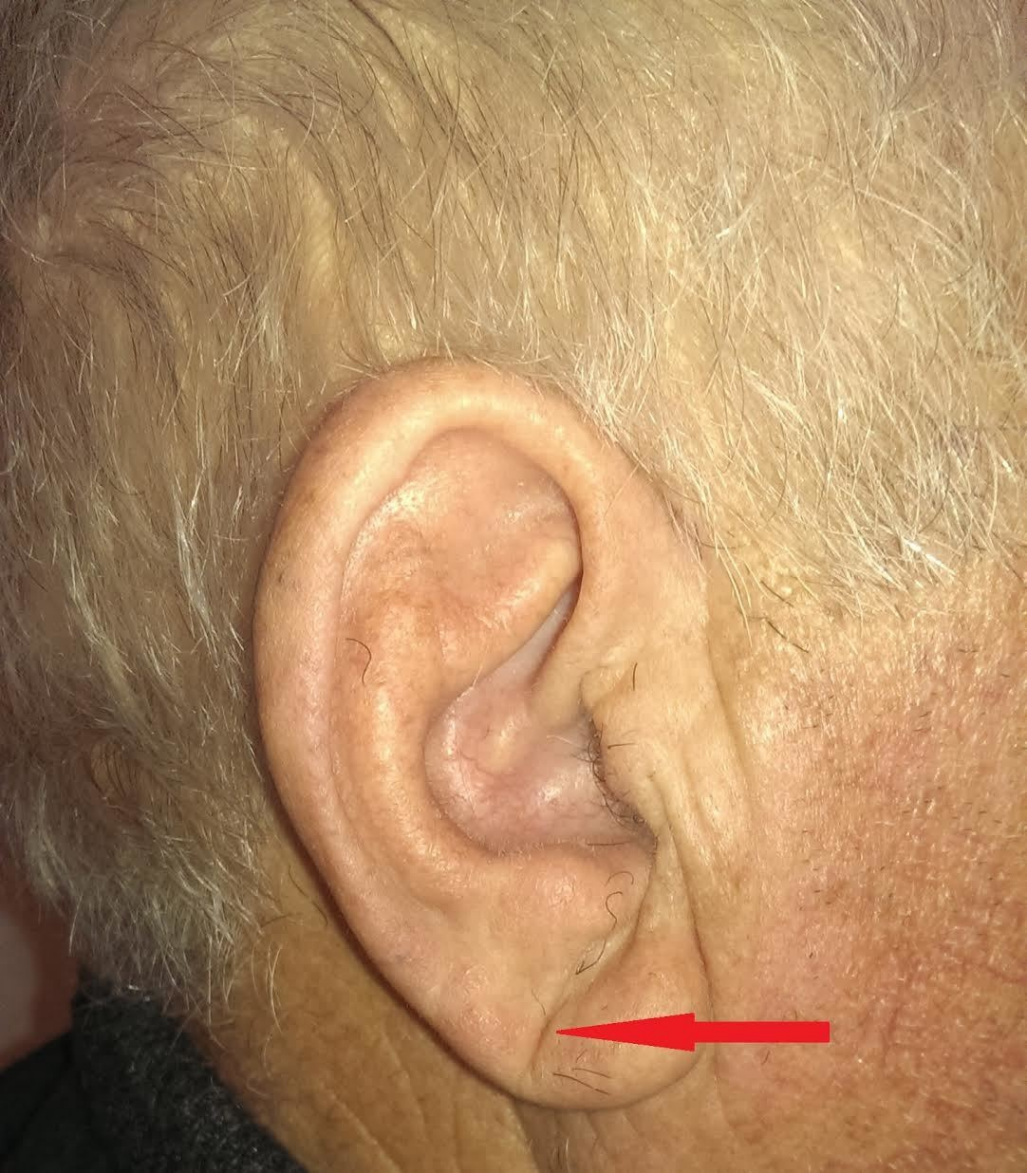
The presence of a DELC is defined as a deep crease or wrinkle present on the earlobe, running from the lower pole of the external meatus, diagonally backwards to the edge of the lobe at approximately 45 degrees without discontinuity covering at least 2/3rd of its path

Patients were examined for the presence of DELC of the lobular portion of either auricle. Patients were made to sit upright in bed and both ears were exposed and examined in adequate light. The examination was performed separately by two different examiners in an attempt to account for any bias or inter-examiner variability. The presence of a DELC was defined as a deep crease or wrinkle present on the earlobe, running from the lower pole of the external meatus, diagonally backward to the edge of the lobe at approximately 45 degrees without discontinuity covering at least 2/3rd of its path (Figure 1).

The presence of DELC on both ears was documented as bilateral and presence of crease on any one of the ears was considered unilateral. Patients with ear piercings that could confuse the presence of DELC were excluded from the study. Data was subsequently entered in the statistical package 16 and analyzed. Comparison between the cases and controls was carried out using chi-square test. Odds ratios (OR) with 95% confidence intervals were calculated using an online statistical software package for two-way contingency table analysis.

## Results

**Figure 2:**
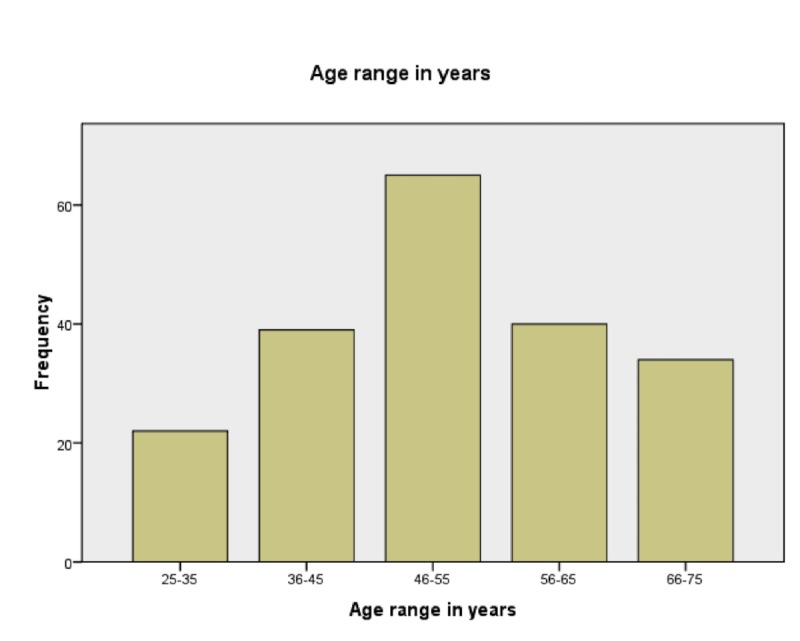
Frequency of age and ranges of the participants

A total of 200 patients (100 cases, 100 controls), aged between 25-75 years, of which 126 were males and 74 were females were enrolled in this study (Figure 2) (Table 1).

 

**Table 1:** Clinical features of patients included in the study

Variables	Cases (n=100)	Controls (n=100)	P values
Males	66	60	0.464
Females	34	40	0.464
Smokers	27	25	0.747
Hypertensives	66	27	0.001
Diabetics	53	18	0.001

The group of patients who had established CAD (cases) consisted of 66 males and 34 females (n=100), of which 76 had DELC and 24 had no DELC (p= 0.001, OR = 5.63, CI = 2.917-10.938), showing a significant association. Bilateral crease was present at a higher frequency among cases than controls (p=0.001; OR =11.429; CI=5.102-26.03). Unilateral crease was observed at a higher frequency among the controls. However, it was not significant (p=0.102; OR=1.939; CI= 0.8-4.6) (Table 2).

**Table 2:** Occurrence of diagonal earlobe crease in cases and controls Abbreviations-DELC: diagonal earlobe crease, CI: confidence interval

		DELC present	Bilateral DELC	Unilateral DELC	No DELC
Cases	Number	76	60	16	24
(n=100)	Frequency	0.76	0.60	0.16	0.24
Controls	Number	36	14	22	64
(n=100)	Frequency	0.36	0.14	0.22	0.64
	P-value	<0.00	<0.00	<0.102	
	Odds ratio	5.63	11.429	1.939	
	95% CI	2.917-10.938	5.102-26.03	0.811-4.64	

Thus the odds of development of CAD in a patient with DELC, bilateral or unilateral is 5.63 times greater than the patient without DELC. The sensitivity was 0.76, specificity 0.64, the positive predictive value 0.678 and negative predictive value 0.727. Of the total 52 smokers, only 19 had the bilateral crease (p=0.895). Of the total hypertensive (n=93), a significant 46 had bilateral DELC (p=0.003). And out of the total diabetics (n=71), 43 had bilateral DELC (p=0.001). On the basis of these results, the effect of hypertension and diabetes on the presence of DELC was statistically significant (p <0.05). However, smoking did not show any significant correlation with the presence of DELC (p >0.05) (Table 3).

**Table 3:** Clinical features of patients correlated with the occurrence of diagonal earlobe crease Abbreviations-DELC: diagonal earlobe crease

Variables	Bilateral DELC	Unilateral DELC	No DELC	P value
Smokers	19	11	22	0.895
Hypertensives	46	15	32	0.003
Diabetics	43	14	14	0.001

## Discussion

Since the first report of Frank in 1973, the association of CAD with DELC has been a matter of debate amongst researchers [3]. Several studies [4,8] in the past have shown a positive association of DELC with CAD, a finding corroborated with the results of our study. It is proposed that the crease is an external sign of a microangiopathic lesion of terminal vessels that occurs in systemic diseases such as CAD. An evaluation of 338 patients with documented evidence of CAD showed a significant association with DELC, with sensitivity and specificity of 65% and 72% respectively [10]. In another study done in California, 430 patients were assessed for association between DELC and CAD using coronary computed tomography angiography. DELC was found in 71% of patients with CAD, concluding the importance of earlobe crease as an independent marker predicting the prevalence, extent, and severity of CAD [11]. In proposition, a review article discussed the benefit of DELC as a valuable marker for atherosclerotic risk assessment [12].

However, some studies [12-13] have failed to substantiate this relationship and denied any correlation between DELC and CAD contrary to the results obtained in our research. Some studies attribute the occurrence of DELC as a sign of ageing, being overweight and hyperuricemia, thus negating any relevance to CAD [14]. This brings up the challenge and need for further studies to be conducted to reach a broader consensus towards this important relationship and also to explore other factors potentially playing a role towards this association.

The role of race and ethnicity has been proposed in the association between DELC and CAD, however, this has been minimally investigated [9]. In a study performed in the Turkish population, 415 cases showed a statistically significant prevalence of DELC [51.4%]. These were patients with a positive angiogram (> 70% stenosis of the luminal diameter) compared to those with normal angiograms (15.1%) [15]. On the contrary, a Japanese study regarded DELC solely as a physical sign of ageing rather than a predictor of CAD by determining shorter mean terminal telomerase fragment in peripheral blood cells of Japanese participants with metabolic syndrome [16]. These conflicting results indicate the variability of this association in different races and perhaps the role of ethnicity as a driving factor in deciphering the importance of DELC as a predictive marker for CAD. Our study, conducted in a South Asian population shows a significant association between patients suffering from cardiovascular diseases and DELC.

When taken into consideration, our study also investigated the role of hypertension, diabetes, and smoking in the development of an earlobe crease. These associations have been studied a few times in the past but with conflicting results. Evrengul, et al. described a positive correlation of DELC with CAD, hypertension, age, male gender and cigarette smoking in a study and suggested that bilateral DELC could be a dermatological sign and a diagnostic tool in clinical examination of patients [15]. Our study corroborates these results as in concluding a positive correlation of hypertension and the presence of DELC. On the contrary, no association was found in a case-control study between the occurrence of DELC and any risk factor such as smoking, hypertension, diabetes and weight in another study done by Jorde, et al. [17]. Limited literature is available to prove the relationship of diabetes and earlobe crease. Theoretically, sustained hemodynamic actions on the microvasculature occurring in diabetes results in increased capillary wall permeability, basement membrane thickening and narrowing of the lumen. The complications eventually engulf the surrounding tissue influencing the blood circulation and functional consequences [18-19]. This theory is supported by our findings of a spastically significant relationship between diabetics and the presence of earlobe crease. The negative association of smoking and DELC in our present data was consistent with some studies. Our study concluded a significant association of hypertension and diabetes in the presence of DELC but no statistically significant role of smoking could be established.

In this study, we confirmed a positive association of earlobe crease with CAD in 100 Pakistani patients with established CAD, using coronary angiography as the gold standard test for CAD (p <0.05). Out of the total number of patients recruited, the presence of DELC was significantly correlated with diabetics and hypertensive patients but not with smokers. The negative association of smoking and DELC in our present data was consistent with findings of some studies [20]. However, one study opposes this conclusion, suggesting a significant positive correlation between smoking and DELC [21]. There is room for more studies to be carried out to establish a conclusion and to better understand the link of DELC and smoking. Our study is the first in South Asia to investigate the roles of hypertension, diabetes, and smoking in the development of DELC.With high-quality data collection and analysis, DELC, inspected upon general physical examination may be effectively used as a marker to predict the risk of development of CAD. Patients can subsequently be counseled for prevention of further exposure to greater risk factors, control of the preexisting risk factors such as hypertension and given prompt treatment to reduce morbidity and mortality. Through this case-control study, we observed the occurrence of diagonal earlobe crease in patients with established coronary artery disease.

### Limitations

Grading for the earlobe crease occurrence in terms of mild, moderate, severe and the severity of coronary artery disease was not done. Recent evidence has suggested that the extent of DELC is proportional to the severity of CAD. Due to resource implications, the controls were only verified for CAD by prior hospital records for no evidence of established CAD. 

## Conclusions

To the best of our knowledge, this study is the first report disclosing the correlation of diagonal earlobe crease with coronary artery disease in Pakistani and South Asian population. We concluded that there is a significant association of diagonal earlobe crease with coronary artery disease. Furthermore, the frequency of diagonal earlobe crease was high in patients with hypertension and diabetes but not in smokers. According to our result, this physical sign could be used as a useful marker for the prediction of coronary artery disease. More prospective cohort studies need to be conducted to confirm this association overcoming ethnic differences in the results.
